# Development and characterization of natural sourced bioplastic for food packaging applications

**DOI:** 10.1016/j.heliyon.2023.e13538

**Published:** 2023-02-06

**Authors:** Mohammad Asaduzzaman Chowdhury, Nayem Hossain, M.D. Badrudduza, Md. Masud Rana

**Affiliations:** a Department of Mechanical Engineering, Dhaka University of Engineering and Technology (DUET), Gazipur, Gazipur, 1707, Bangladesh; b Department of Mechanical Engineering, IUBAT-International University of Business Agriculture and Technology, Bangladesh; c Department of Mechanical Engineering, DUET-Dhaka University of Engineering and Technology, Gazipur, Gazipur, 1707, Bangladesh

**Keywords:** Bioplastic, Food packaging application, Tamarind, Berry, Licorice

## Abstract

Climate change and increased pollution caused by traditional petrochemical plastics made the biodegradable environment-friendly plastic (bioplastic) research more popular. Bioplastics can be manufactured from natural renewable ingredients and used as food packaging material without harming the environment. This research work focuses on developing bioplastic films from natural ingredients such as starch of tamarind seeds, and berry seeds, with licorice root. Attention has been paid to characterizing the material by biodegradability, mechanical testing, Fourier Transformed Infrared Spectroscopy (FTIR), Scanning Electron Microscopy (SEM), Thermogravimetric Analysis (TGA), Differential Scanning Calorimetry (DSC), antimicrobial analysis tests. Phenolic compounds present in the berry seeds starch increased the soil biodegradability as well as the mechanical and thermal properties of the bioplastic films. The FTIR spectra confirmed the presence of various bio-molecules. Improved antimicrobial performance is also obtained. The results of this research confirm that the prepared bioplastic samples can be used in packaging applications.

## 1 Introduction

The increased use of petrochemical plastics has become a great threat to human beings and living organisms' survival due to environmental pollution made by these petrochemical plastics. Emphasis is being paid and a lot of research work is going on to manufacture environmentally friendly biodegradable green plastics to replace the traditional harmful petrochemical plastics [[1], [2], [3], [4], [5]]. Recent years have seen increased research on the development of bioplastics and biocomposites [[6], [7], [8]]. The potentiality available in bioplastics can replace petrochemical plastics in a different home and industrial applications [[9], [10], [11], [12]]. Starch is an abundant, available, and low-cost source of raw material used to make bioplastics [13]. Starch is rich in glucan that has mainly two components namely amylose and amylopectin. Hydroxyl groups are available in starch which makes it hydrophilic. Linear molecules of glucose and bifurcated molecules are available in amylase and amylopectin respectively. Besides, hydrated starch is thermally conductive and biodegradable [14].

Tamarind is an evergreen tree that needs a dry climate to grow. Every part of tamarind is rich in nutritional value and is used as medicine. Its seeds are a good source of protein good for human health [15]. Tamarind seed can be used in paint and cosmetic industries due to its acidic value. Polysaccharides present in tamarind seeds are biodegradable and biocompatible in nature and can be extracted from the endosperm of the seed [16]. Tamarind seed polysaccharide is composed of monosaccharide, octasaccharide, heptasaccharide, hexasaccharide, and hendesaccharide [17]. Films produced from tamarind seeds polysaccharides can remain thermally stable up to the temperature range of 201.88 °C [18].

Berries are good for health due to the presence of antioxidant and organoleptic properties [19]. Berries are rich in phenolic compounds which contain different types of cyaniding, peonidin, etc at different percentages [20]. Berries are effective against bacteria, viruses, fungi, archaebacteria, and protozoa [21]. Besides, berries promote human health and can prevent different diseases such as cancers, neurodegenerative, heart diseases, etc. Bioactive compounds are also present in berries with dietary compounds [22].

Licorice is a valuable medicinal plant that has more than 30 species worldwide and is mainly found in Asia and the Mediterranean region [23]. Licorice root contains different types of bioactive compounds and as a result, it is used as medicine to treat diseases. It is expected that licorice root will be used to treat arthritis, asthma, and colitis. Licorice root contains glabridin which shows antibacterial effects on different types of pathogenic bacteria [24].

Here, the prime objective of this work is to show the usability of the developed bioplastic as a food packaging material to replace traditional petrochemical plastic. The focus was paid to evaluating its biodegradable, mechanical, morphological, thermal, and antibacterial performance.

In this study, two types of bioplastic samples were prepared, with berry seeds starch and without berry seeds starch keeping the licorice root percentages constant. In this study, we used 20 g licorice root. But in the previous study, we used 10 g licorice root. Here, the addition of berries increased biodegradation. Besides, the addition of berries developed mechanical properties. Moreover, the addition of berries increased antimicrobial properties.

## 2 Materials and methods

### 2.1 Materials

Tamarind seeds, berry seeds, licorice root, distilled water, glycerol, and white vinegar were used to fabricate the bioplastic samples. The agricultural lab of the Department of Agriculture of IUBAT provided the necessary seeds of berry and tamarind with licorice. Distilled water was collected from the environment lab of the Civil Engineering Department of IUBAT as well. We collected glycerol and white vinegar from a nearby shop.

### 2.2 Bioplastic sample preparation

**Fig. 1:**
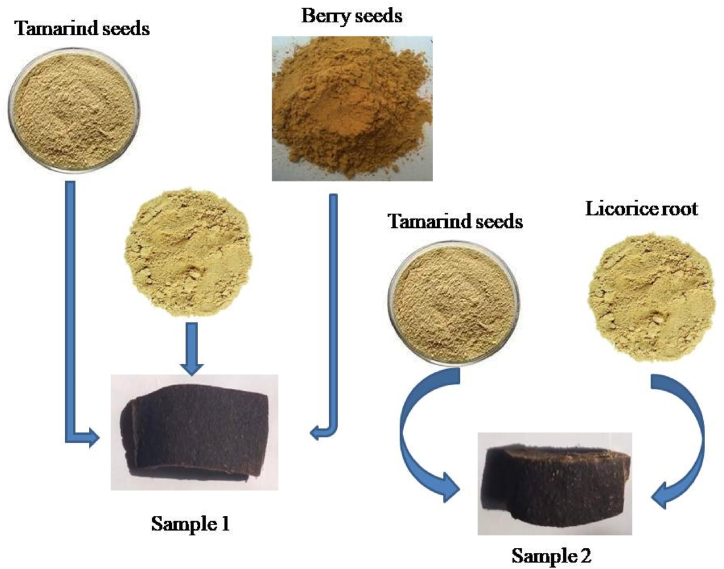
Production of bioplastic films.

Dirt was removed from the solid ingredients by washing three times with distilled water followed by boiling and blending to get starch. Two types of samples were prepared where one sample contained tamarind seeds starch and licorice root and another sample contained starch of berry and tamarind with licorice shown in Fig. 1. The percentages of different ingredients can be seen in Table 1. The ingredients were weighed, mixed, blended, and stirred at 100 °C. Bioplastic was prepared after several minutes of heating which was poured on aluminum foil. The bioplastic films were obtained after several hours of cooling.

**Table 1:** Used raw materials to make bioplastic samples.

Sample	White vinegar (ml)	Glycerol (ml)	Distilled water (ml)	Tamarind seed starch (g)	Berry seed starch (g)	Licorice Root(g)
S1	40	40	360	60	20	20
S2	40	40	360	60	–	20

### 2.3 Characterization

#### 2.3.1 Biodegradability test

The biodegradability of the prepared bioplastic samples was evaluated in the microorganism present soil. The bioplastic samples were cut at the dimension of 4 cm × 2 cm for the test. The prepared bioplastic samples were buried in the soil for different time periods. Each sample was measured for weight by precise electronic balance before burying. Each sample was cleaned carefully by running tap water followed by drying at room temperature under the sun after removing from the soil and weight was taken again. The weight loss data was obtained from the differences between the two values which indicated the soil biodegradability rate of the prepared bioplastic samples [25]. Three measurements were conducted for the samples considered the average value and the corresponding standard deviation was calculated. Weight loss was calculated by the following formula:

(1) $$Weight\;loss\;\left(\%\right)=\frac{W0-W1}{W1}\times 100$$

Here, W0 and W1 are the weights before and after test [[26], [27], [28], [29], [30], [31], [32]].

#### 2.3.2 Mechanical test

ASTM-D638-14 standard was maintained to perform the mechanical test of the prepared bioplastic samples by a computer-controlled CMT-10 electronic universal testing machine. The experiments were conducted at room temperature at a 2 mm/min strain rate. The prepared samples for the mechanical test had 165 mm overall length with 57 mm length of the narrow section and 50 mm gage length. The narrow section had a 13 mm width and the overall thickness was 3.5 mm. Five experiments were conducted for each sample, the average value was considered and the corresponding standard deviation was calculated.

#### 2.3.3 FTIR test

The FTIR profile from the bioplastic samples was obtained from Perkin Elmer Spectrometer. The presence of different functional groups was identified using the wave number ranging from 500 cm^−1^ to 4000 cm^−1^.

#### 2.3.4 SEM test

The microstructure of the bioplastic films was analyzed by Hitachi S-4800 scanning electronic microscope. The influence of the berry seeds extract on the structure generated was determined. The samples were cut into 0.5 cm^2^ size, immersed in liquid nitrogen, curio fractured, randomly broken, and coated with gold palladium. The samples were observed at a 10 mm working distance, 10 kV accelerating voltage and different magnifications.

#### 2.3.5 Thermal analysis

TA SDT 650 instrument was employed to perform TGA and DSC analysis of the bioplastic films at a weight rate of 10–25 mg, a heating rate of 5 °C, and from room temperature to 650 °C.

#### 2.3.6 Antimicrobial assay

ASTM E2149-01 standard was followed to analyze the antimicrobial properties of the bioplastic films against the gram-positive *S. aureus* and gram-negative *E. coli* bacteria in the disk diffusion method.

## 3 Results and discussion

### 3.1 Soil biodegradation performance analysis

**Fig. 2:**
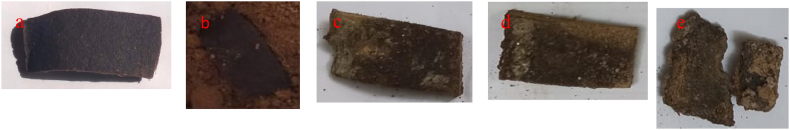
Soil biodegradability of the sample S1 after (a) 0 days (5 gm), (b) 7 days (14), (c) 15 days (20), (d) 21 days (40), (e) 30 days (60).

**Fig. 3:**
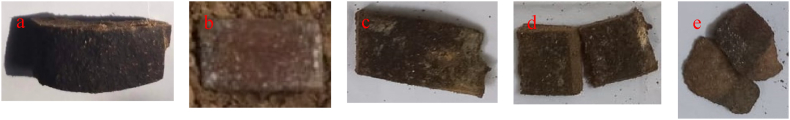
Soil biodegradability of the sample S2 after (a) 0 days (5 gm), (b) 7 days (10), (c) 15 days (18), (d) 21 days (38), (e) 30 days (46).

**Fig. 4:**
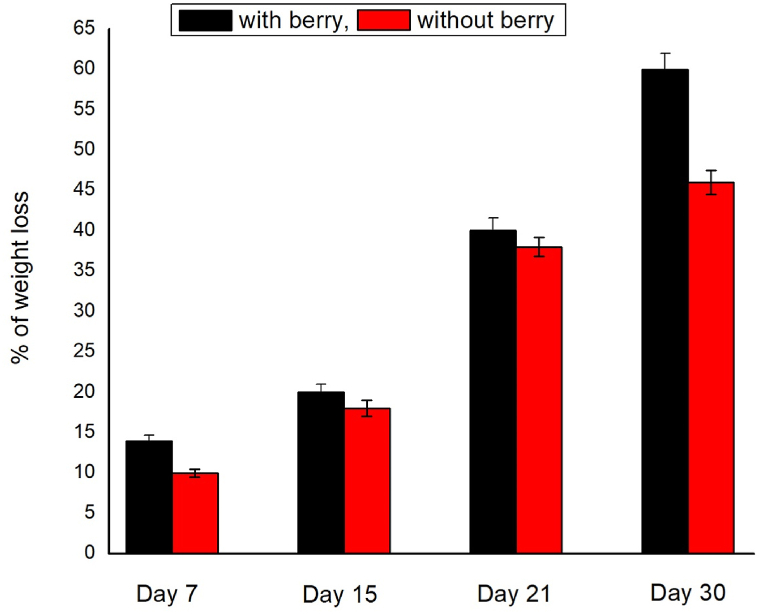
Biodegradability comparison of the prepared bioplastic samples.

Fig. 2 (a to e) and 3 (a to e) shows the soil biodegradability of the prepared bioplastic samples with time variation. Fig. 4 shows the biodegradability comparison. From the beginning of soil burial, biodegradation was observed. After 7, 14, 21, and 30 days of observation, 14%, 20%, 40% and 60% biodegradation was observed from the sample S1. However, for the same time period, 10%, 18%, 38%, and 46% biodegradation were observed for Sample S2. The presence of microorganisms along with their microbial performance is the key to biodegradation [33,34]. Biodegradability increased due to the incorporation of berry starch with other ingredients. The phenolic compounds of berries are mainly responsible for fast biodegradation. Phenolic compounds are biodegradable in nature. It can be converted into quinone cross-linked to protein which later forms a new covalent cross-link at higher pH. A covalent bond in C–N is formed due to the reaction between quinones and amino polypeptides [35]. Besides, phenolic compounds are degraded to carbon dioxide by fermentation [36].

### 3.2 Mechanical properties analysis

**Fig. 5:**
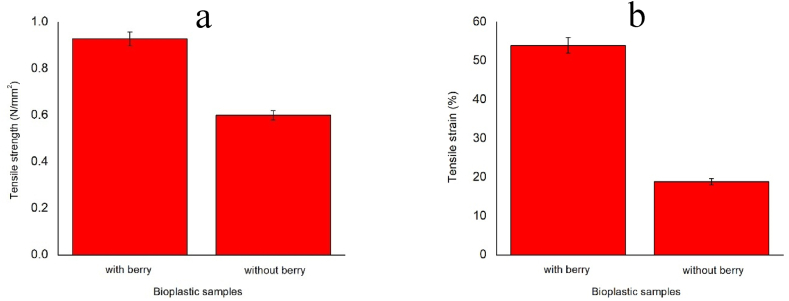
Tensile properties comparison of the bioplastic films (a) tensile strength, (b) tensile strain.

Fig. 5 shows the comparison in tensile strength of the developed bioplastic samples. Better tensile strength and tensile strain were obtained from the bioplastic sample after incorporating berry seeds starch which improved the mechanical properties. The interaction between the phenolic compound and protein is the reason for mechanical improvement which made the bioplastic sample more flexible. A stronger and stiffer film structure was formed due to the presence of phenolic compounds [37]. Ionic, covalent, hydrogen, and hydrophobic bonds are formed by phenolic compounds in the sample. A C–N covalent bond is formed due to berry seeds starch with free amino groups which are available in protein-forming quinones which results in a degree of protein cross-linking gradual increase. The mechanical properties are developed by the formation of a typical cross-link. Strong intermolecular connections reduced the free space of the polymer matrix which made the bioplastic sample stiffer, denser, and tightly packed with reduced molecular mobility [38,39]. Cross-links can be formed by the phenolic compounds among the proteins that react with several protein sites [40].

### 3.3 Morphological analysis

**Fig. 6:**
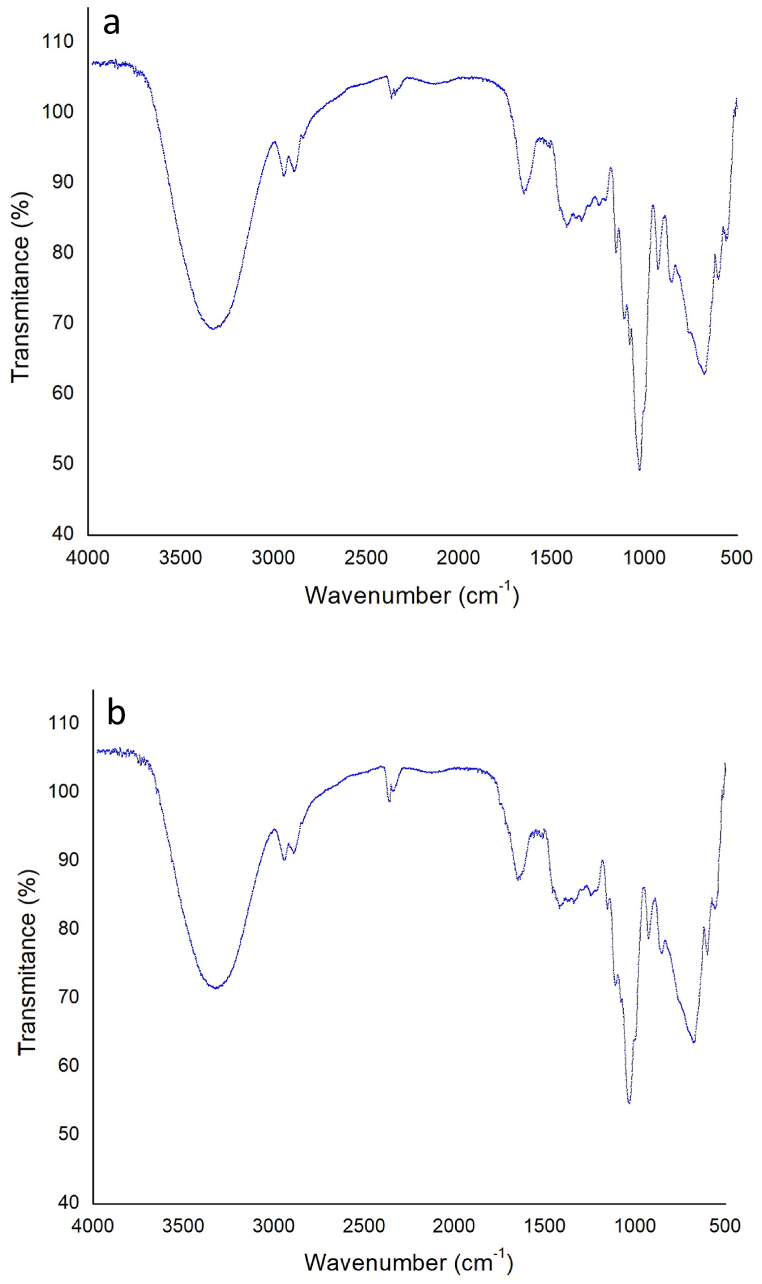
FTIR analysis of the synthesized bioplastic (a) with berry seeds and (b) without berry seeds.

Fig. 6 (a and b) shows the FTIR spectra of the prepared bioplastic samples. The FTIR spectra show the presence of different functional groups in the prepared samples. The presence of berry, licorice root, and tamarind was confirmed by the characteristic peaks of the spectra. Similar characteristic peaks were observed from the bioplastic samples because of having similar constituents but at different intensities. The presence of licorice increases the intensity of the spectra. Medium stretching aliphatic primary amine N–H was identified by the broad absorption peak at 3325 cm^−1^. The intensity of N–H peaks shifted to 3323 cm^−1^ with greater intensity after the addition of berry seeds starch which signifies the excellent dispersion of berry seeds starch in the films. The peak at 2889 cm^−1^ is attributed to the medium stretching alkane C–H confirming the presence of polysaccharides [41]. The peak is shifted to 2943 cm^−1^ with berry seeds starch addition. Peaks in sample S2 at 2357 cm^−1^ denote strong stretching carbon dioxide CO_2_ which is shifted to 2362 cm^−1^ in sample S1. Peaks at 1647 cm^−1^ represent strong stretching Imine C–N. Peaks at 1417 cm^−1^ are attributed to medium stretching carboxylic acid OH which is shifted to 1409 cm^−1^ after berry seeds starch addition which indicates the formation of inter and intermolecular hydrogen bonds and confirms the presence of glabridin [42,43]. Medium bending amine group was identified at 1029 cm^−1^ by the absorption band. Sample S1 identified the presence of C–Br at 675 cm^−1^ and S2 at 677 cm^−1^. Table 2 shows various functional groups identified by the FTIR spectra.

**Table 2:** Presence of various functional groups identified by the FTIR spectra.

Band (cm^−1^)	Functional class	Assignment	Vibration type
With Berry seeds			
675	Halo compound	C–Br	Strong stretching
1029	Amine	C–N	Medium bending
1409	Carboxylic acid	O–H	Medium bending
1647	Imine	C=N	Medium stretching
2362	Carbon dioxide	O=C=O	Strong stretching
2943	Alkane	C–H	Medium stretching
3323	Aliphatic primary amine	N–H	Medium stretching
Without Berry Seeds			
677	Halo compound	C–Br	Strong stretching
1029	Amine	C–N	Medium bending
1417	Carboxylic acid	O–H	Medium bending
1647	Imine	C=N	Medium stretching
2357	Carbon dioxide	O=C=O	Strong stretching
2889	Alkane	C–H	Medium stretching
3325	Aliphatic primary amine	N–H	Medium stretching

**Fig. 7:**
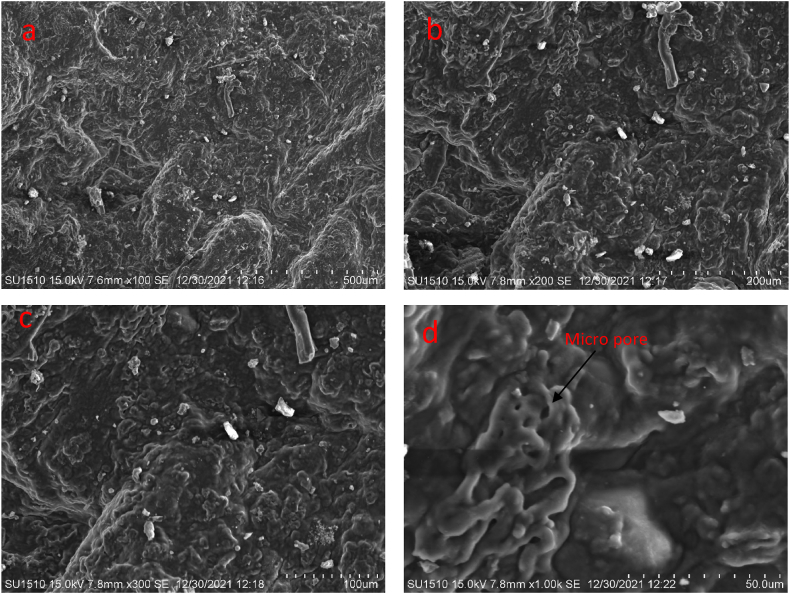
SEM surface morphology of the bioplastic film prepared with berry seeds starch at (a) 500 μm and (b) 200 μm, (c) 100 μm, and (d) 50 μm.

**Fig. 8:**
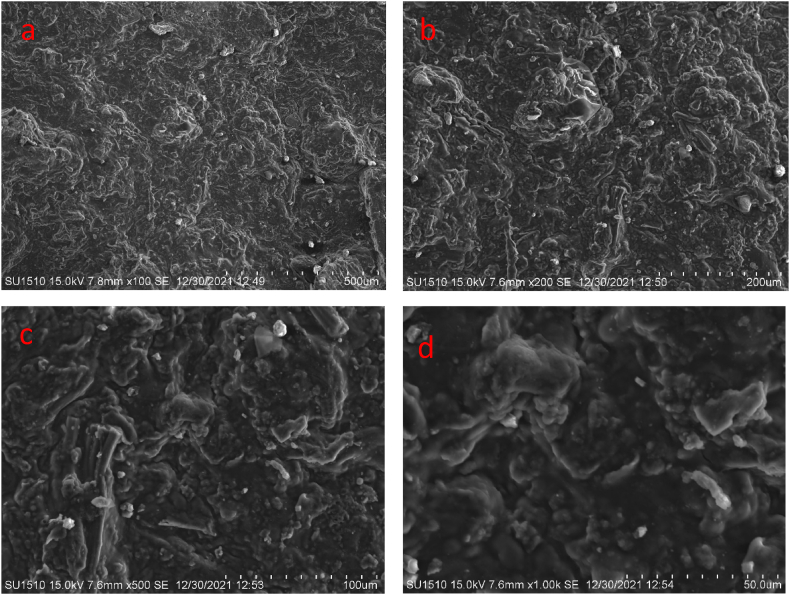
SEM surface morphology of the bioplastic film prepared without berry seeds starch at (a) 500 μm and (b) 200 μm, (c) 100 μm, and (d) 50 μm.

Fig. 7 (a to d) and 8 (a to d) shows the SEM surface morphology of the developed bioplastic samples. The previously observed results and the observed results by SEM analysis show similarities. Similar observations were found through the addition of berry seeds starch. A good number of micro-pores are observed from the analysis which helps to biodegrade the bioplastic samples interacting with the microorganism available in soil [44]. Both samples show good surface integrity and a homogeneous phase. Coarse, granules, irregularities, and flaws can be seen on the surface of both of the samples. The presence of foreign particles is observed on the surface of both samples.

### 3.4 Thermal analysis

#### 3.4.1 TGA analysis

**Fig. 9:**
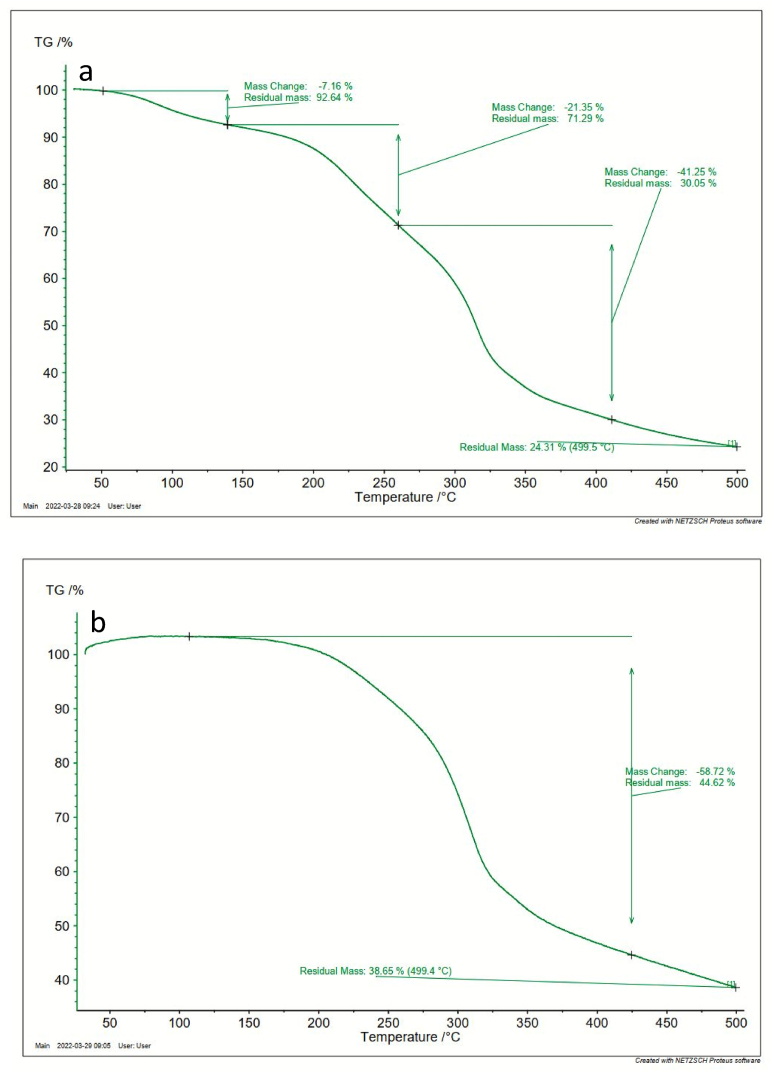
TGA graph of the prepared bioplastics samples (a) with berry seeds starch, (b) without berry seeds starch.

**Fig. 10:**
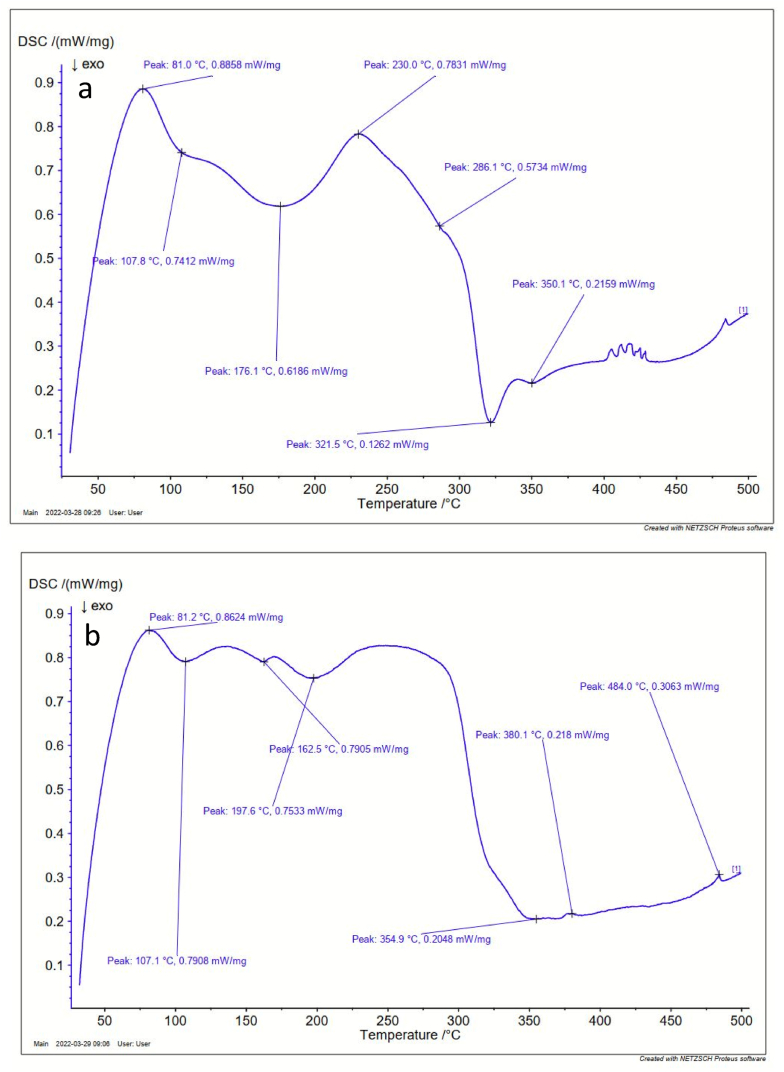
DSC graph of the prepared bioplastics samples (a) with berry seeds starch, (b) without berry seeds starch.

The thermal stability of the developed bioplastic samples prepared with and without berry seeds starch was analyzed by the Thermogravimetric Analysis (TGA) shown in Fig. 9 (a to b) and Differential Scanning Calorimetry (DSC) shown in Fig. 10 (a to b). Weight losses for sample S1 were observed at three different periods. 130 °C, 260 °C, and 410 °C. Evaporation of water made the first weight loss at 130 °C [45]. Phenolic compounds decompose thermally which made the second weight loss at 260 °C. At 200 °C, salvianolic acid as well as ferulic acid available at phenolic compounds decomposes. At 250 °C, decomposition occurs of protocatechuic acid present in the phenolic compound [46]. Starch degrades at 410 °C which is the third degradation [47]. On the other hand, only one weight loss is observed at sample S2 nearly at 425 °C as sample S2 does not contain phenolic compounds.

#### 3.4.2 DSC analysis

Exothermic and endothermic curves of the developed bioplastic samples analyzed by DSC are shown in Fig. 10 (a and b). The first exothermic peak determined the glass transition temperature which is also termed starch gelatin temperature. Good mechanical properties with flexibility can be obtained from developing bioplastic at this temperature from starch. At this temperature, the mechanical properties of the prepared bioplastic films change from elastic to brittle due to changes in chain mobility. The incorporation of berry seeds starch resulted in minimal change in this parameter. The results indicate that this temperature is suitable for bioplastic casting. Glass transition temperature was observed at 81.0 °C for sample S1 and 81.2 °C for sample S2. Moreover, glass-like characteristics can be observed in terms of strength, brittleness, and stiffness from the developed bioplastic samples as the glass transition temperature was observed above room temperature [48]. Besides, above the glass transition temperature, the bioplastic polymer chains have high mobility and can form ordered arrangements due to having high energy as well as undergoing crystallization.

### 3.5 Antibacterial performance analysis

**Fig. 11:**
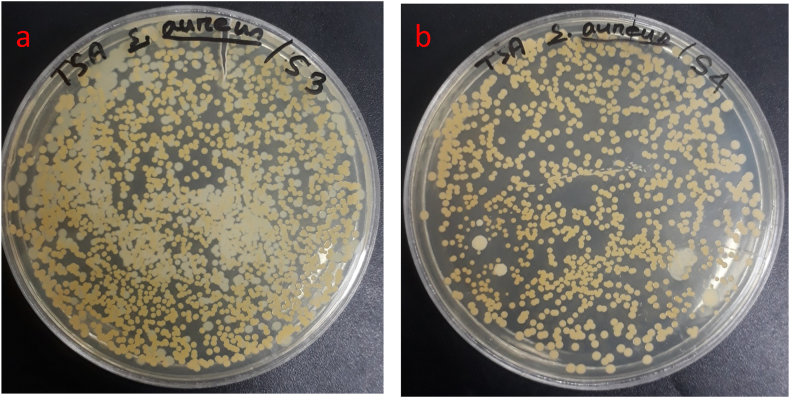
Antibacterial reduction performance of the prepared bioplastic samples (a) with berry seed starch and (b) without berry seeds starch.

**Fig. 12:**
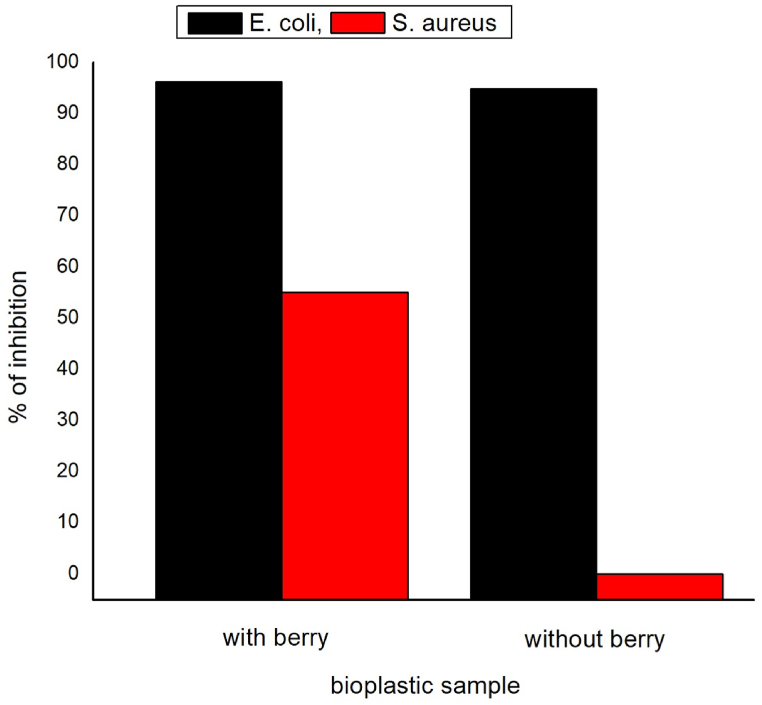
Bacterial reduction performance comparison.

Fig. 11 (a and b) and 12 show the antibacterial performance of the developed bioplastic samples against both gram-positive and gram-negative bacteria strain *S. aureus* and *E. coli* respectively. *E. coli* is naturally available in food items and the environment. It causes diarrhea, sepsis, and urinary infections [49]. *S. aureus* causes various clinical infections such as skin and soft tissue-related infections, pleuropulmonary and pleuropulmonary [50]. Bacterial inhibition was characterized as % for the developed bioplastic samples. Sample S1 showed better bacterial inhibition against both bacteria strains. However, both samples worked better against *E. coli*. A thick polypeptide layer having a thickness of 55 nm without any outer lipid membrane is possessed by the gram-positive bacteria which is difficult to break. On the other hand, a thin polypeptide layer having only a 2 nm thickness wall with an outer lipid membrane is possessed by the gram-negative bacteria which is easy to disrupt and kill the bacteria strain [51]. Moreover, Phenolic compounds show good antibacterial properties against bacterial strains [52]. That is why; the incorporation of berry seed extracts increased the antibacterial strain of the synthesized bioplastic films.

The prepared bioplastic samples are the ideal candidate for food packaging applications as this material posses the characteristics that can improve foods' shelf life. It can be said from the results that samples S1 possesses characteristics that can protect the food improving the physical barrier and blocking bacterial pass [[53], [54], [55], [56]].

## 4 Conclusion

Fabrication and characterization of bioplastic from natural sources for food packaging applications have been reported here. Berry seeds starch was incorporated with tamarind seeds starch and licorice root to develop the bioplastic samples. Improved soil biodegradable, tensile, antimicrobial, thermal, and morphological performance was observed from the prepared bioplastic samples. The properties improved due to the increased crosslinking of berry seeds starch with other materials. The incorporation of berry seeds starch improved the properties remarkably and behaved similarly to the materials used for food packaging applications. Therefore, the results indicate that the prepared bioplastic films especially sample S1 can be used as biodegradable alternatives for food packaging applications. Other natural starch can be used to improve the mechanical properties of bioplastic films.

## Author contribution statement

Mohammad Asaduzzaman Chowdhury: Conceived and designed the experiments.

Nayem Hossain: Analyzed and interpreted the data; Wrote the paper.

Md. Badrudduza: Performed the experiments.

Md. Masud Rana: Contributed reagents, materials, analysis tools or data.

## Funding statement

This research did not receive any specific grant from funding agencies in the public, commercial, or not-for-profit sectors.

## Data availability statement

Data will be made available on request.

## Declaration of interest’s statement

The authors declare no conflict of interest.
